# The Inhibition of Histone Lysine Demethylases Affects Head and Neck Cancer Cell Growth and Response to Cisplatin

**DOI:** 10.3390/genes17091047

**Published:** 2026-08-30

**Authors:** Dawid Dorna, Robert Kleszcz, Violetta Krajka-Kuźniak, Jarosław Paluszczak

**Affiliations:** 1 Poznan University of Medical Sciences, Doctoral School, 60-812 Poznań, Poland; dawid.dorna97@gmail.com; 2 Poznan University of Medical Sciences, Department of Pharmaceutical Biochemistry, 60-806 Poznań, Poland; kleszcz@ump.edu.pl (R.K.); vkrajka@ump.edu.pl (V.K.-K.)

**Keywords:** head and neck cancer, histone lysine demethylase, KDM4, KDM5, KDM6, ML324, GSK-J4, JIB-04, cisplatin

## Abstract

**Background/Objectives**: Head and neck squamous cell carcinomas (HNSCC) are a heterogenous group of tumors, which are usually treated with surgery and radiotherapy, while chemotherapy (e.g., cisplatin), anti-EGFR, or immune checkpoint inhibitors are used to treat locoregionally advanced and recurrent cases. However, survival outcomes are still unsatisfactory, and novel augmenting treatments are required. Recently, histone lysine demethylases (KDMs) emerged as promising anti-cancer targets. In order to further characterize KDM4–6 as pharmacological targets in HNSCC, we evaluated changes in KDM4/5/6 expression in HNSCC patients, and we experimentally assessed the biological effects of inhibitors of KDM4/5/6 in HNSCC cell lines. **Methods**: We used online tools (TIMER, GEPIA3, UALCAN) to analyze TCGA data from HNSCC patients. Then, we used resazurin assay to assess the effect of ML324, GSK-J4, and JIB-04 (the inhibitors of KDM4, KDM6, and KDM4–6, respectively) on cell viability in two-dimensional (2D) and three-dimensional (3D) culture of FaDu, SCC-152, and Detroit-562 cells. The potential for cisplatin sensitization was also assessed. Gene/protein expression was evaluated by qPCR and antibody array. **Results**: We found that the majority of the analyzed KDMs were overexpressed in HNSCC, except *KDM4C*, *KDM5D*, and *KDM6A*. All three studied inhibitors significantly reduced cell viability in 2D and 3D cell cultures. Moreover, the pre-treatment of cells with KDM inhibitors showed moderate potential for cisplatin sensitization. In addition, the chemicals significantly reduced the expression of pluripotency markers, especially SOX2. **Conclusions**: In conclusion, KDM4–6 inhibitors significantly affected HNSCC cell growth, and exerted the potential for epigenetic priming in combination with cisplatin.

## 1. Introduction

Head and neck squamous cell carcinoma (HNSCC) is the seventh most prevalent type of cancer, with over 940,000 new cases, and 480,000 deaths noted globally in 2022 [1,2,3]. This heterogenous group consists of tumors located in the lip, oral cavity, larynx, pharynx (divided into nasopharynx, oropharynx, hypopharynx), salivary glands, nasal cavity and paranasal sinuses. Etiologically, it is associated with long-term tobacco smoking, betel quid chewing, and frequent consumption of alcohol. Moreover, viral infections with human papillomavirus (HPV) or Epstein–Barr virus (EBV) contribute to the development of oropharyngeal and nasopharyngeal cancers, respectively [4]. Importantly, HPV-positive cases showed better prognosis and more favorable outcomes than HPV-negative HNSCC patients [5]. The mutation of *TP53* is the most frequent genetic alteration observed in both HPV-negative and HPV-positive HNSCC, while other molecular changes are more heterogeneously distributed among HNSCC patients [5,6]. Surgery and radiotherapy are most frequently used for HNSCC treatment. Furthermore, chemotherapy (cisplatin, 5-fluorouracil, docetaxel) can be applied for the treatment of locally advanced or unresectable tumors, and targeted therapy against epithelial growth factor receptor (cetuximab) or immune checkpoint inhibitors (pembrolizumab, nivolumab) can be used in advanced, recurrent and metastatic HNSCC cases [7,8]. However, patients diagnosed at advanced stages still show unfavorable prognosis (shorter overall and progression-free survival), despite progress in diagnostic and therapeutic strategies [3,7]. Therefore, ongoing efforts focus on the development of novel and more effective treatments or treatment combinations.

Epigenetic abnormalities are prevalent in cancer cells, which are characterized by altered profiles of DNA methylation and histone modifications, especially histone acetylation and methylation. So far, DNA methyltransferase (DNMT) and histone deacetylase (HDAC) inhibitors have been used clinically to restore the expression of epigenetically silenced tumor suppressor genes, but other epigenetic writers, readers, and erasers, have also emerged as promising molecular targets [9,10,11].

Reversible histone methylation plays many important functions in cellular regulation, including modulation of gene expression. Methylation of histone H3 lysine 4 (H3K4) or H3K36 promotes transcription, while H3K9 and H3K27 methylation blocks transcription. The level and profile of these marks is regulated by specific histone methyltransferases and histone lysine demethylases (KDMs). KDMs from the Jumonji C family (KDM2–8) have been shown to significantly contribute to carcinogenesis in many types of cancer, including HNSCC, by affecting cell signaling, metabolic reprogramming, cell proliferation and cell death, stemness, cell motility, tumor microenvironment, and immune response [12,13,14]. The modulation of stemness potential was found to be one of the key mechanisms of the anti-cancer action of KDM inhibition, causing the elimination of tumor-initiating stem-like cells [14,15]. It has been hypothesized that the eradication of cancer stem cells could significantly support long-term HNSCC control and survivorship outcomes [16]. Moreover, KDM1 inhibitors showed more selective effects on cancer-related gene expression profiles, in comparison to other epigenetic drugs (e.g., HDAC inhibitors), This suggests that KDM inhibition may cause fewer side effects in patients, although this hypothesis still requires validation [17]. Furthermore, several reports indicated that KDM4/5/6 were associated with response to chemotherapeutics, including cisplatin, but evidence referring to head and neck cancers is scarce [18,19,20]. In our recent study, we have shown that KDM inhibitors (KDMi) targeting KDM4 (ML324), KDM6 (GSK-J4), or KDM4–6 (JIB-04) reduced HNSCC cell viability and promoted apoptosis in HNSCC cells; however their co-administration with cisplatin did not lead to significantly enhanced effects. On the other hand, triple combinations consisting of ML324 or JIB-04 together with cisplatin and olaparib—a PARP1 inhibitor which affects DNA damage response—led to synergistic enhancement of apoptosis induction, partly by disrupting DNA repair [21]. Sequential treatments may offer an advantage over concurrent administration. Indeed, the concept of epigenetic priming has been coined to describe the triggering of changes in the profile of epigenetic modifications which enable further biological effects mediated by additional treatments. For example, the pre-treatment of gastric cancer cells with decitabine, a DNMT inhibitor, sensitized cells to cisplatin and SN38 (the active metabolite of irinotecan), but not paclitaxel or 5-fluorouracil [22]. In addition, 5-azacytidine, a DNMT inhibitor, and entinostat, an HDAC inhibitor, increased sensitivity of lung cancer xenografts to irinotecan, but not cisplatin [23].

Thus, one of the aims of the present study was to evaluate whether pre-treatment of HNSCC cells with KDM4–6 inhibitors could increase cellular response to cisplatin. In addition, we wanted to validate the effects of the studied KDMi and their combinations with cisplatin in a more relevant three-dimensional (3D) model of cells grown as spheroids. Indeed, we observed that ML324 and JIB-04 were able to enhance to some extent the response of HNSCC cells to cisplatin. In addition, we wanted to further verify the relationship between KDM expression and the expression of key stemness genes, to better understand the pharmacology behind the anti-cancer activity of KDM4–6 inhibitors.

## 2. Materials and Methods

### 2.1. The Analysis of the Cancer Genome Atlas Data

Gene expression data from HNSCC patients and relevant non-cancer controls, which are available from The Cancer Genome Atlas (TCGA), were analyzed using several online tools. TIMER v.3 (https://compbio.cn/timer3/, accessed on 15 May 2026) [24] was used to evaluate the changes in the level of expression of individual KDMs in HNSCC patients, in comparison to non-cancer controls. TIMER3 was also used for the analysis of the correlation between the expression of individual KDMs and stemness-related genes. TIMER v.2 (https://compbio.cn/timer2/, accessed on 12 May 2026) [25] was used for the analysis of the changes in KDM expression in HPV-positive and negative HNSCC patients. It also served for the analysis of the association between KDM expression and patients’ overall survival. In addition, GEPIA v.3 (https://gepia3.bioinfoliu.com/, accessed on 17 June 2026) [26] was used to analyze the association between KDM expression levels and overall survival in HNSCC patients, using the median expression level as the cutoff value. The UALCAN tool (https://ualcan.path.uab.edu/, accessed on 17 June 2026) [27,28] was applied for the evaluation of the association between KDM expression and *TP53* mutation status or clinical stage.

### 2.2. Cell Lines and Culture Conditions

Three HPV-negative (FaDu, Detroit-562, UM-SCC-1) and one HPV-positive (SCC-152) HNSCC cell lines were used in this study. The FaDu cell line was derived from a primary hypopharyngeal tumor. The Detroit-562 cell line was derived from a metastatic pharyngeal tumor. The SCC-152 cell line was derived from a recurrent hypopharyngeal tumor, which was primarily located in the base of the tongue. These cell lines were provided from the American Type Culture Collection (ATCC, Manassas, VA, USA). UM-SCC-1 cell line was derived from a tumor located in the floor of the mouth. It was purchased from Merck (Darmstadt, Germany).

All cell lines were cultured in complete medium composed of high-glucose DMEM (Biowest, Nuaillé, France) supplemented with 5% Fetal Bovine Serum (EURx, Gdańsk, Poland), and 1% antibiotics solution (penicillin and streptomycin, Biowest, Nuaillé, France). Three-dimensional cell spheroids were cultured in Nunclon Sphera ultra-low attachment plates (ThermoFisher Scientific, Waltham, MA, USA) in a medium composed of high-glucose DMEM supplemented with 2% B27 and 1% N2 (ThermoFisher Scientific, Waltham, MA, USA). Cells were grown at standard conditions: 37 °C, 90% humidity, and 5% CO_2_.

### 2.3. Chemicals

In the study, three KDM inhibitors (KDMi) were used: GSK-J4 (targeting KDM6), ML324 (targeting KDM4), and JIB-04 (targeting KDM4-6). Additionally, the platinum-based antineoplastic agent cisplatin was tested. All chemicals were purchased from MedChemExpress (Monmouth Junction, NJ, USA). KDM inhibitors were dissolved in DMSO and stored in aliquots at −20 °C, while cisplatin stock solutions were prepared in saline and stored in light-protected tubes at room temperature up to one month.

### 2.4. Cell Viability Evaluation

To evaluate the effects of the tested compounds and their combinations on cell viability, the resazurin assay was employed. For the 2D assay, cells (6.0 × 10^3^ cells/well) were seeded into 96-well plates. After 24 h, the cells were treated with the tested compounds or their combinations, while control wells received the vehicle (DMSO). Following 72 h of incubation, the culture medium was removed, the cells were washed with warm PBS, and resazurin solution (1 μg/mL in PBS; Sigma-Aldrich, St. Louis, MO, USA) was added to each well. After 90 min of incubation, fluorescence was measured. In the case of sequential treatments, cells were initially seeded into 100 mm plates, and treated with the indicated concentrations of KDMi on the following day. After 3 days of treatments with KDMi, cells were collected by trypsinization and seeded into 96-well plates. On the following day, cells were treated with the indicated range of cisplatin concentrations. After 3 days, viability was assessed using the resazurin assay. Relevant experimental setups are presented in each respective figure.

For the 3D assay, cells (5.0 × 10^3^ cells/well) were seeded into Nunclon Sphera 96-Well U-Shaped-Bottom Microplates (Thermo Fisher Scientific, Waltham, MA, USA). After four days of spheroid formation, the tested compounds were added in a fresh portion of culture medium. Following 72 h of treatment, resazurin solution was added to each well to achieve a final concentration of 1 μg/mL. After an additional 24 h of incubation, fluorescence was measured. In the case of sequential treatments, cells were seeded into ultra-low attachment plates, and KDMi were added after 48 h of spheroid formation. Following another 48 h, cisplatin was added into wells. After a subsequent 4 days of spheroid growth, viability was evaluated using the resazurin assay. For reliable results in a 3D system, resazurin was added 24 h before fluorescence measurements.

Each time, fluorescence was measured using an Infinite M200 multimode microplate reader (Tecan, Grödig, Austria) at excitation and emission wavelengths of 530 and 590 nm, respectively. Each experiment was performed independently at least three times for every treatment condition, with several (3–6) technical replicates per assay. Blank measurements were always included. After blank subtraction, relative viability upon treatment with inhibitors was calculated as the percentage of the signal measured in control wells (with cells exposed to the same amount of vehicle).

In the majority of combination treatments, compounds were applied at their IC25 concentrations, with additional conditions using half the IC25 or twice the IC25. IC values were determined using either a four-parameter logistic (4PL) curve fit or linear interpolation, depending on the shape of the dose–response curve and the biological plausibility of the resulting concentration. IC50 values could not always be reliably determined, particularly in 3D models, and therefore could not be used as a standardized measure of equipotency across compounds.

### 2.5. Gene Expression and Protein Network Analysis

Detroit-562 cells (1.5 × 10^5^ cells/well) were seeded into 6-well plates and treated with the indicated concentrations of the chemicals for 72 h. Next, RNA was isolated using RNA Extracol (EURx, Gdańsk, Poland), and reverse transcribed using smART First Strand cDNA Synthesis Kit (EURx, Gdańsk, Poland). The relative level of expression of pluripotency genes (*ABCG2*, *ALDH1A1*, *NANOG*, *POU5F1*, *SOX2*) was evaluated using SG qPCR Master Mix (EURx, Gdańsk, Poland). Two housekeeping genes—*TBP* and *PBGD*—served as reference genes for the calculation of relative change in gene expression level using the ΔΔCt method. Data from three independently performed experiments were analyzed. Amplification conditions and starter sequences were previously described [21,29,30]. The following sequences were used for the assessment of *NANOG* expression: forward—5′CCAGGATTTTAACGTTCTGCT, reverse—5′TTTTGCGACACTCTTCTCTG.

The protein interaction network between KDMs and stemness regulatory proteins was evaluated using STRING v.12.0 (https://string-db.org/, accessed on 17 June 2026) [31].

### 2.6. Pluripotency Protein Content Analysis Using an Antibody Array

Cells were seeded into 100 mm plates at a density of 1.0 × 10^6^ cells per well in 10 mL of DMEM supplemented with 5% FBS and 1% antibiotics. After 24 h, the culture medium was replaced with 10 mL of fresh medium containing the indicated KDM inhibitors. Following 72 h of treatment, total cellular protein was isolated. The culture medium was aspirated, and the cells were washed once with PBS. Lysis buffer supplemented with protease inhibitors (Halt Protease Inhibitor Cocktail, 100×, Thermo Fisher Scientific, Waltham, MA, USA) was added, and the cells were scraped on ice. The lysates were transferred into low-protein-binding tubes, incubated on a rocker for 30 min at 4 °C, and subsequently centrifuged at 14,000× *g* for 5 min at 4 °C. The resulting supernatants were collected, and protein concentrations were determined using the Pierce BCA Protein Assay Kit (Thermo Fisher Scientific, Waltham, MA, USA). Equal amounts of total protein (250 μg) were analyzed using the Proteome Profiler Human Pluripotent Stem Cell Array Kit (Bio-Techne, Minneapolis, MN, USA) according to the manufacturer’s instructions. Following chemiluminescent detection, the membranes were imaged using a Bio-Rad ChemiDoc imaging system, and the signal intensity of each spot was quantified using Image Lab software, version 6.1.0 (Bio-Rad, Hercules, CA, USA). Two independent biological experiments were performed.

### 2.7. Statistical Analysis

The Student’s *t*-test was used to compare the experimental groups with the control. Differences between treatments with individual compounds and their combinations were analyzed using the one-way ANOVA followed by Tukey’s post hoc test. Differences were considered statistically significant at *p* < 0.05.

## 3. Results

### 3.1. Histone Lysine Demethylases 4–6 Are Overexpressed in HNSCC Patients

**Figure 1:**
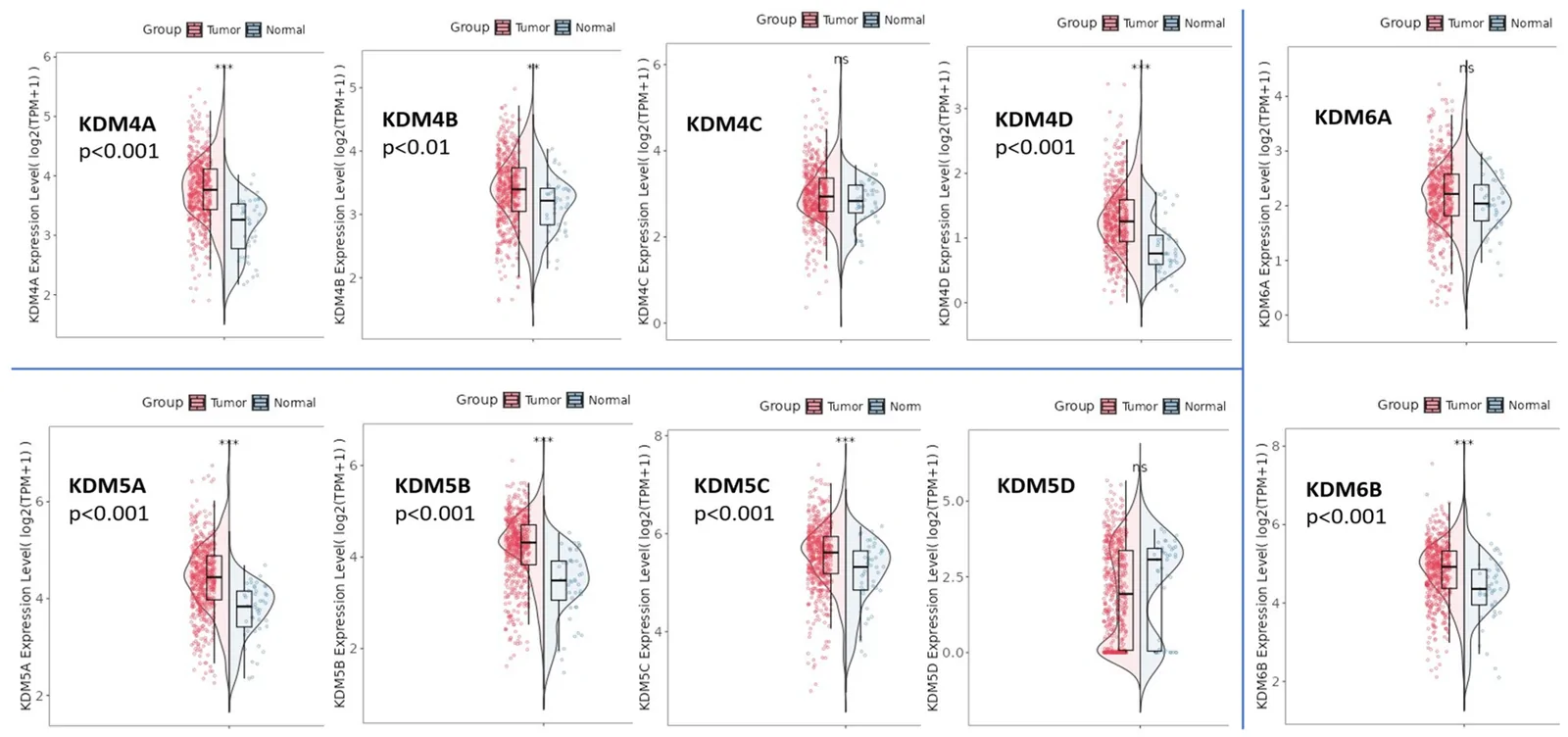
The expression of *KDM4-6* isoforms in HNSCC patients from the TCGA dataset. The analysis was performed using TIMER3. Statistically significant changes in the level of expression between HNSCC tumor cases (*n* = 520, red, left) and non-tumor samples (*n* = 44, blue, right) are noted with *p* values beneath respective KDM symbols, and asterisks above bars (** *p* < 0.01, *** *p* < 0.001, and ns = not significant).

In the first step, we evaluated changes in the level of expression of KDMs in HNSCC patients, in comparison to non-cancer controls. The analysis of the TCGA data using the TIMER3 tool showed statistically significant overexpression of *KDM4A*, *KDM4B*, *KDM4D*, *KDM5A*, *KDM5B*, *KDM5C*, and *KDM6B* in HNSCC patients (Figure 1). The expression of *KDM4A-D*, *KDM5A*, *KDM6A* tended to be higher in HPV-positive cases, in comparison to HPV-negative HNSCC patients. The opposite trend was noted in the case of *KDM5B* and *KDM6B* (Supplementary File S1). The overexpression of *KDM4A-D* and *KDM5A–C* was observed in all HNSCC cases irrespective of the presence or absence of *TP53* mutations. Furthermore, *KDM6A* was significantly overexpressed only in patients without *TP53* mutations, while *KDM6B* was overexpressed only in patients showing the presence of *TP53* mutations, in comparison to normal control samples (Supplementary File S1).

**Figure 2:**
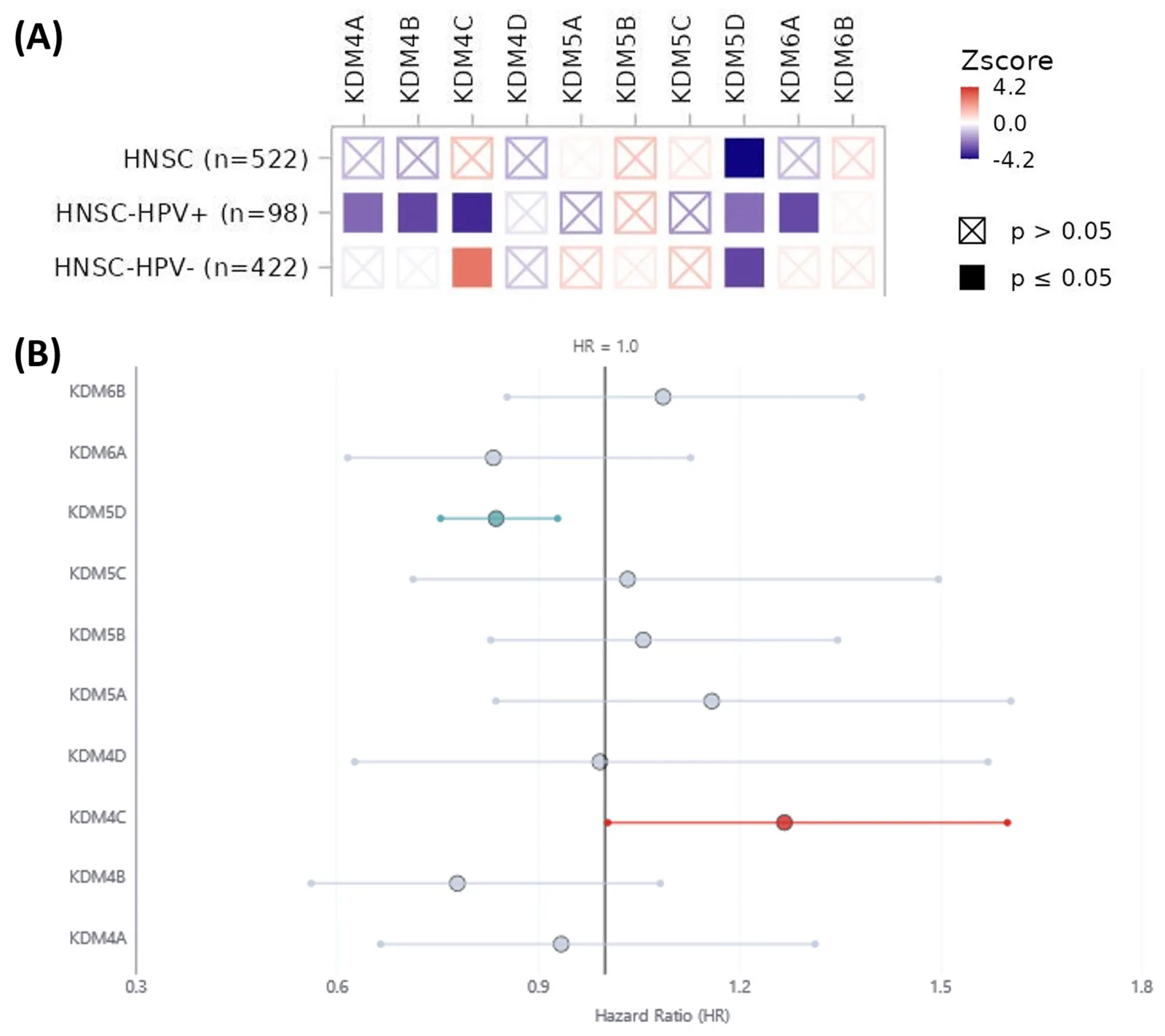
The association between KDM expression level and overall survival in HNSCC patients. (**A**) The results of the Cox proportional hazard ratio analysis performed using TIMER2.0, where blue and orange correspond to longer, and shorter OS, respectively, (**B**) the results of multivariate analysis performed using GEPIA3.

Next, we wanted to assess the prognostic significance of KDMs. We analyzed the association between the level of expression of KDMs and overall survival (OS) in HNSCC patients using the GEPIA3 tool. We found that higher expression of *KDM4A*, *KDM4B*, and, most profoundly, *KDM5D* was associated with longer OS (Table 1). The expression of other KDMs was not associated with survival. Respective Kaplan–Meier survival plots are presented in Supplementary File S2. Moreover, the Cox proportional hazard model performed using TIMER2.0 pointed to significant association of *KDM5D* with better survival in HNSCC patients, irrespective of HPV status (Figure 2A). In addition, higher expression of *KDM4A*, *KDM4B*, and *KDM6A*, was associated with longer survival in HPV-positive HNSCC patients. Interestingly, the overexpression of *KDM4C* was associated with longer survival in HPV-positive cases, in contrast to HPV-negative patients, in whom it was associated with unfavorable prognosis. These observations were partly supported by the multivariate analysis performed using GEPIA3, which pointed to the significant association between higher *KDM4C* expression and shorter OS, and between higher *KDM5D* expression and better prognosis in HNSCC patients (Figure 2B).

### 3.2. The Expression of KDM4–6 Correlates with the Expression of Stemness Markers

In our previous study, we showed that KDMi successfully downregulated the expression of stemness-related genes (*POU5F1*, *SOX2*, *ALDH1A1*, *ABCG2*) in FaDu cells, and, to a lesser extent, in SCC-152 cells [30]. Indeed, STRING analysis pointed to several interactions between KDM4C, KDM5B, KDM6A, KDM6B, and selected pluripotency factors, mainly SOX2 and OCT4/POU5F1 (Supplementary Figure S1).

To support these observations, we analyzed the correlation between KDM4-6 expression and stemness markers in HNSCC patients using the TIMER3 tool (Table 2). In the total cohort of HNSCC patients (*n* = 520), all KDM isoforms (except *KDM5B*) were positively correlated with the expression of at least four out of the five analyzed stemness markers (Table 2A). Similar correlations were observed in the group of HPV-negative HNSCC cases (*n* = 421), where only *KDM5D* and *KDM5B* showed positive correlations with the expression of fewer genes (three or two, respectively) (Table 2B). In contrast, fewer positive correlations between KDMs expression and stemness marker expression were noted in HPV-positive cases, and the expression of *KDM5B* and *POU5F1* was negatively correlated (Table 2C).

**Figure 3:**
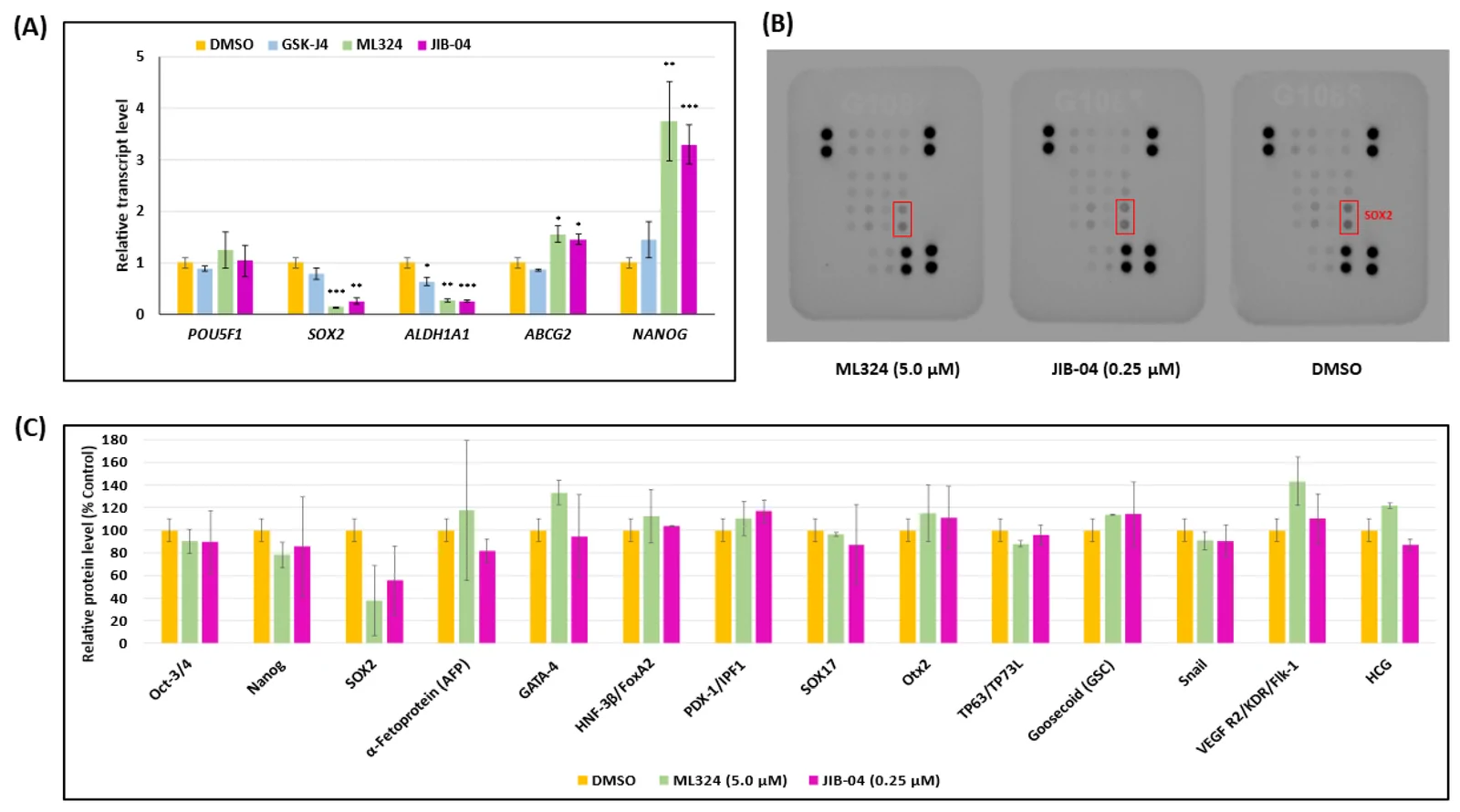
The effect of KDM inhibitors on stemness gene and protein expression in Detroit-562 cells after 72 h. (**A**) The effect of GSK-J4, ML324, JIB-04 at IC25 concentrations on relative mRNA expression of stemness-related genes. Mean values from three biological experiments, with three technical repeats per experiment, are shown ±SD. An asterisk (*) above the bar indicates a statistically significant difference compared with the DMSO control (transcript level = 1), * *p* < 0.05, ** *p* < 0.01, *** *p* < 0.001. (**B**) Representative images of antibody array membranes for the evaluation of the effect of JIB-04 (0.25 µM) or ML324 (5.0 µM) on stemness-associated protein level. (**C**) Relative protein levels expressed as the percentage of the DMSO control. Mean values from two biological experiments, with two technical repeats per experiment, are shown ±SD.

We further experimentally tested the influence of the analyzed KDMi on the expression of key stemness-related genes in Detroit-562 cells, which were derived from a metastatic pharyngeal tumor (Figure 3A). GSK-J4 only moderately decreased the relative transcript level of *ALDH1A1*, with no impact on the expression of other genes. On the other hand, ML324 and JIB-04 presented gene-dependent effects. While *POU5F1* expression remained unaffected, the expression of *SOX2* and *ALDH1A1* was potently reduced, whereas the expression of *ABCG2* and *NANOG* was increased upon 72 h treatment with these compounds.

Next, we evaluated the changes in the protein level of 15 stem cell markers after the treatment of Detroit-562 cells with ML324 and JIB-04 using a dedicated antibody array kit (Figure 3B). We omitted GSK-J4 because of its lack of effect on mRNA levels. No significant changes in protein expression were detected; however, the levels of SOX2 appeared to be reduced following treatment with ML324 and JIB-04 (Figure 3C).

### 3.3. Inhibitors of KDM4–6 Reduce Cell Viability in HNSCC Cell Lines

In order to evaluate the effects of the studied KDMi against HNSCC cell lines which have not been tested so far, we initially performed a 72 h viability assessment of KDM inhibitors (GSK-J4, ML324, JIB-04) and cisplatin in Detroit-562, SCC-152, and UM-SCC-1 cells cultured under conventional 2D conditions. We have previously reported the effects of the studied KDMi on cell viability in FaDu cells [21].

**Figure 4:**
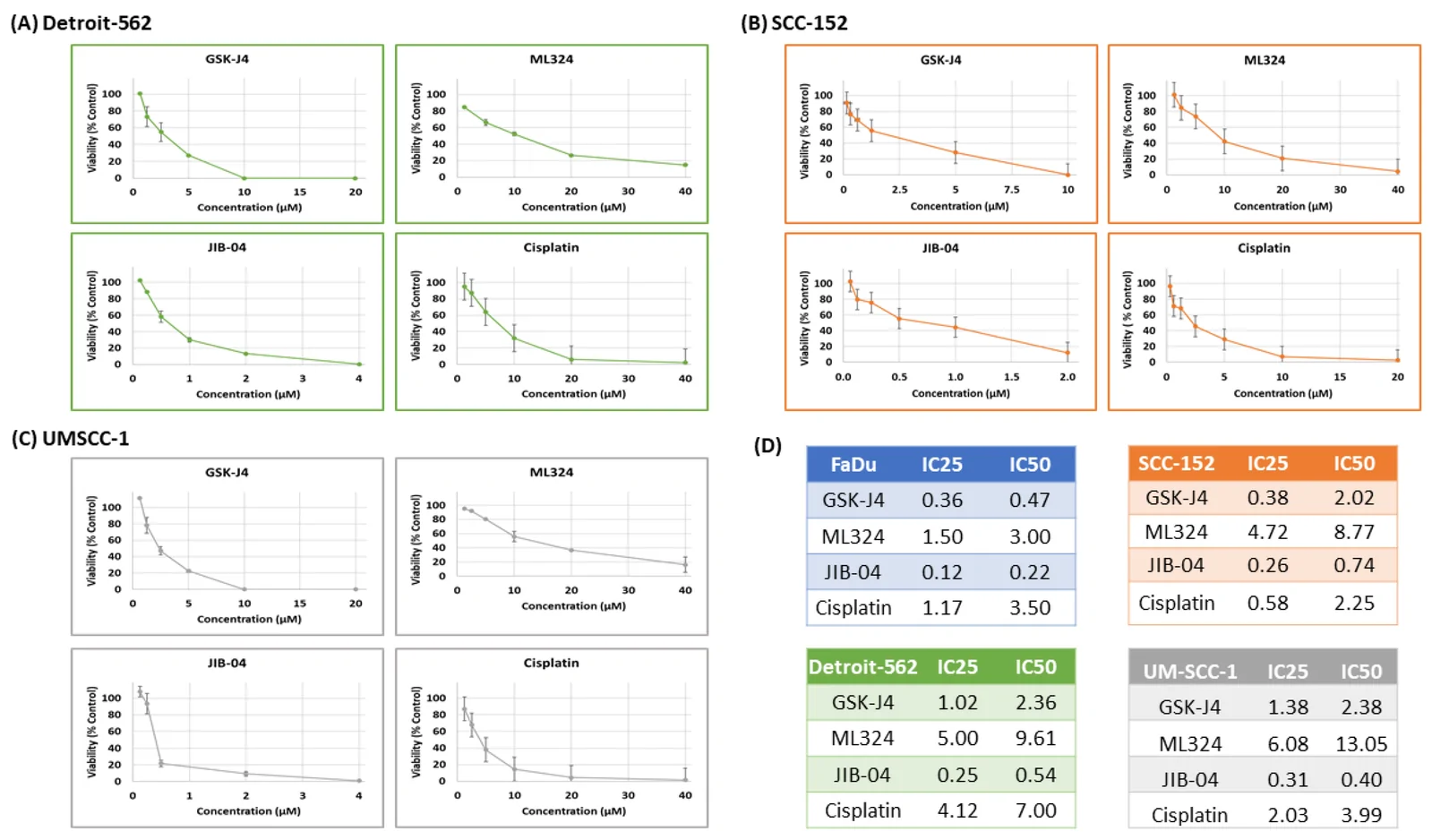
The effect of KDM inhibitors (ML324, GSK-J4, JIB-04), and cisplatin, on cell viability in HNSCC cell lines cultured in 2D for 72 h: (**A**) Detroit-562, (**B**) SCC-152, (**C**) UM-SCC-1, (**D**) values of IC25 and IC50. Mean values from three biological experiments, with four technical repeats per experiment, are shown ±SD.

All the inhibitors potently reduced cell viability across all the studied cell lines (Figure 4). GSK-J4 (KDM6 inhibitor) presented the lowest IC50 value in SCC-152 cells (2.02 µM) (Figure 4B). However, at 10 µM, it completely inhibited viability in all cell lines. Similarly, ML324 (KDM4 inhibitor) most potently reduced the viability of SCC-152 cells at concentrations up to 40 µM. JIB-04 (KDM4-6 inhibitor) demonstrated the greatest efficacy at decreasing cell viability across all tested cell lines, with IC50 values in the submicromolar range (Figure 4D). The activity of cisplatin differed among the cell lines, with the lowest IC50 value (2.25 µM) observed in SCC-152 cells (Figure 4B), and the highest (7.00 µM) in Detroit-562 cells (Figure 4A).

### 3.4. Inhibitors of KDM4–6 Partly Improve Cisplatin-Related Decrease in HNSCC Cell Viability

The main aim of this study was to assess the potential to improve cisplatin’s efficacy against HNSCC cells through additional treatment with KDM4-6 inhibitors. Our earlier report showed that the concurrent treatment of HNSCC cells with KDMi and cisplatin did not lead to improved effects [21]. Thus, we tested sequential treatments in this study.

**Figure 5:**
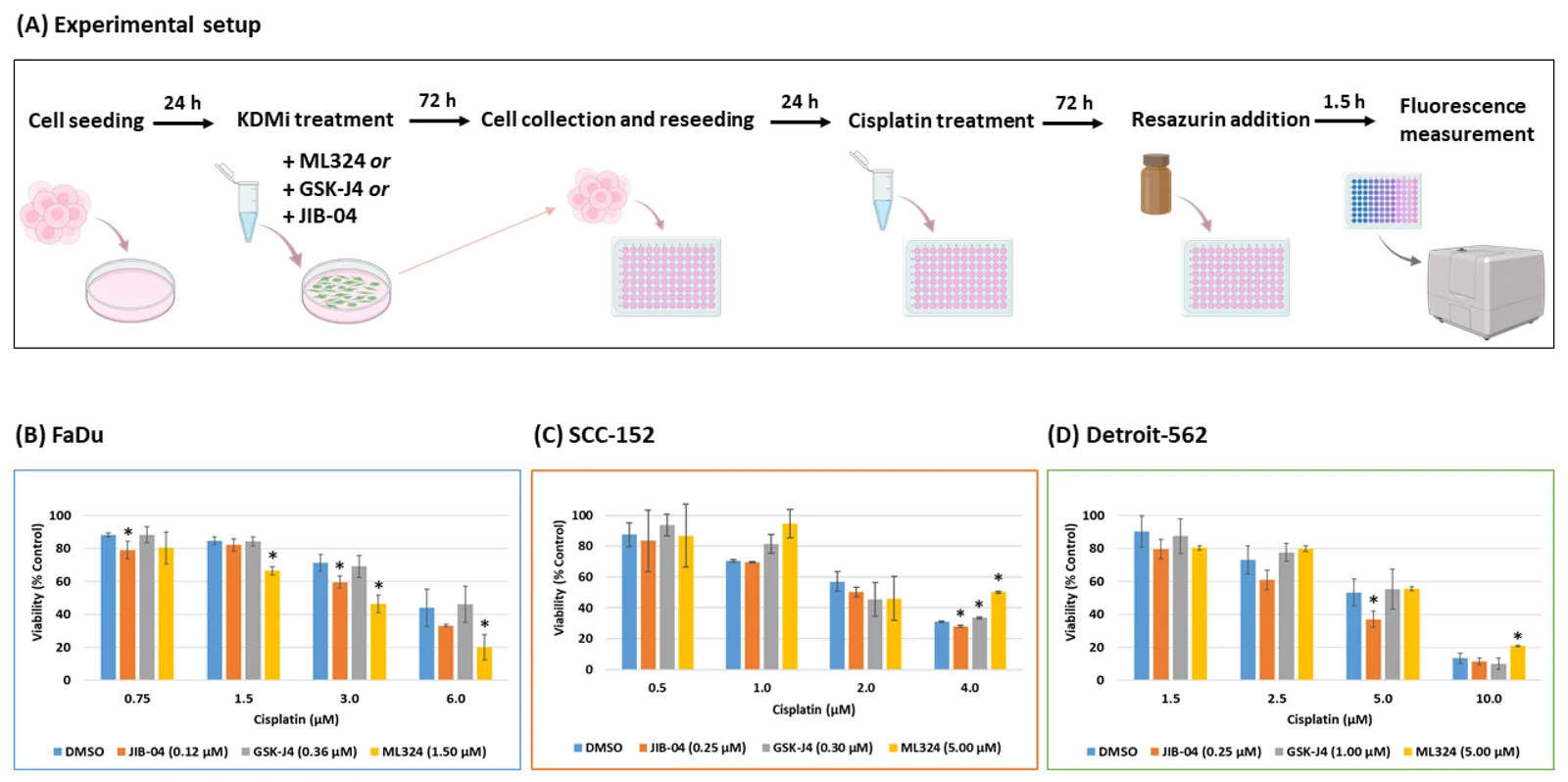
The effect of cisplatin on the viability of cells subjected to pre-treatment with KDM inhibitors in a 2D model in FaDu (**B**), SCC-152 (**C**), and Detroit-562 (**D**) cells. The experimental setup is presented in panel (**A**). Mean values from three biological experiments, with four technical repeats per experiment, are shown ±SD. An asterisk (*) above the bar indicates a statistically significant difference compared with the DMSO control (viability = 100%), * *p* < 0.05.

The initial experiment was performed in a 2D model. FaDu, SCC-152, and Detroit-562 cells were pre-treated with KDMi for 72 h at concentrations close to IC25, reseeded, and treated with cisplatin for an additional 72 h (Figure 5A). Control cells were treated with vehicle.

Several significant reductions in cell viability (*p* < 0.05) were detected in the monolayer culture of HNSCC cells. FaDu cells pre-treated with JIB-04 showed increased response to cisplatin at 0.75 µM and 3.0 µM, while FaDu cells pre-treated with ML324 showed enhanced effects of cisplatin at 1.5 µM, 3.0 µM, and 6.0 µM (Figure 5B). In SCC-152 cells, only pre-treatment with JIB-04 helped to improve the activity of cisplatin at 4.0 µM (Figure 5C). A similar effect for JIB-04 pre-treatment was observed in Detroit-562 cells (Figure 5D).

### 3.5. Inhibitors of KDM4–6 Reduce Cell Viability in HNSCC Spheroids

Three-dimensional cell culture models better reflect the biological conditions of tumor growth in the human body, and they better reflect drug response in tumor cells [32]. Therefore, we performed further experiments using HNSCC cells grown as 3D spheroids.

Assuming that the modulation of stemness potential significantly contributes to the anti-cancer potential of KDM4–6 inhibitors, we optimized the growth conditions in the 3D model. The choice of medium composition for the growth of HNSCC cell spheroids was based on the enrichment in the level of expression of several pluripotency markers: *POU5F1*, *SOX2*, *ALDH1A1*, and *NANOG* (Supplementary Figure S2) Other authors also showed that medium composition may significantly affect the level of expression of stemness markers (especially *POU5F1*, *SOX2*, and *CD90*) in HNSCC spheroids [33]. We applied these conditions for the analysis of the effects of KDMi on viability in HNSCC spheroids.

**Figure 6:**
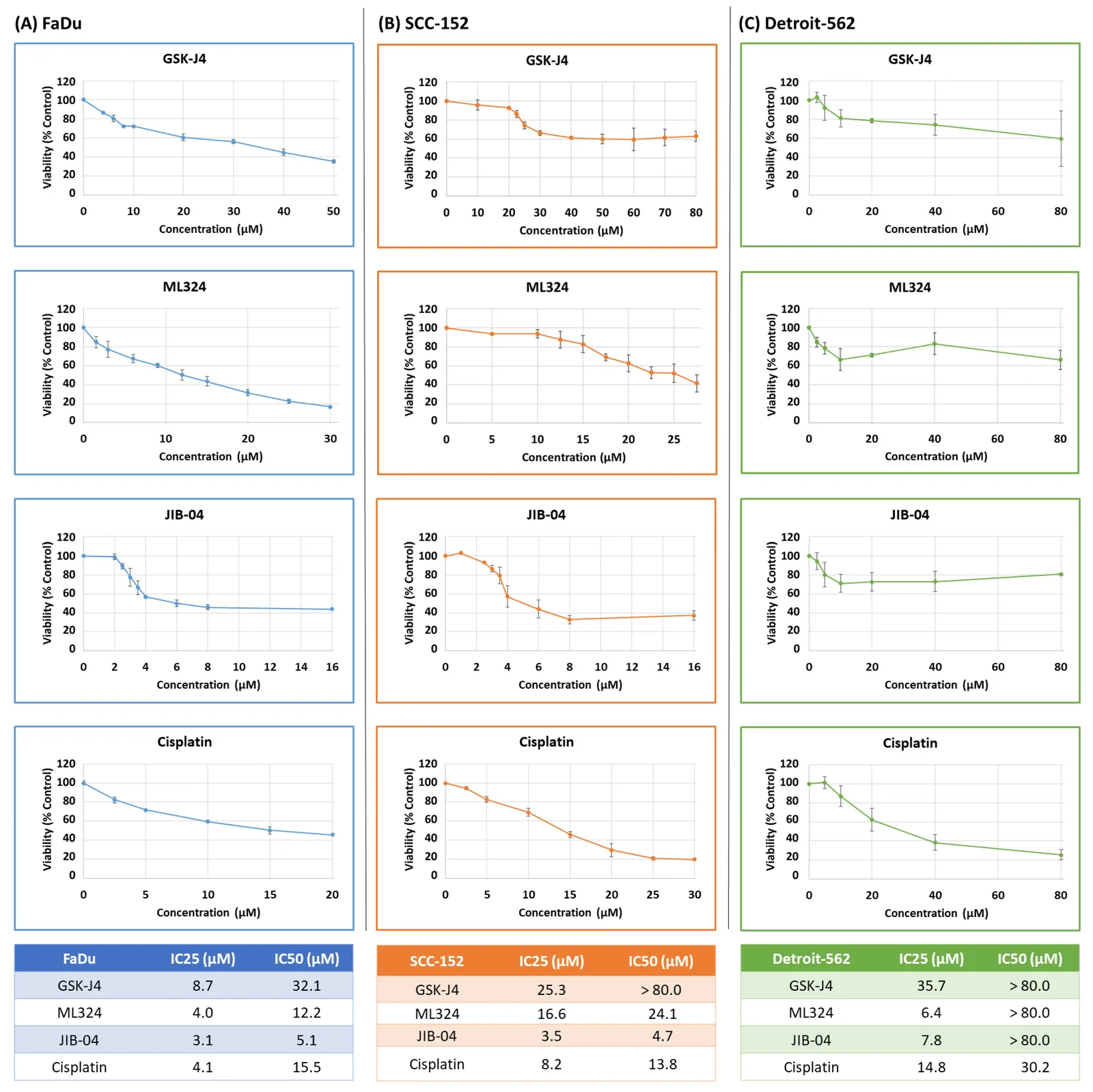
The effect of KDM inhibitors (ML324, GSK-J4, JIB-04), and cisplatin, on cell viability in HNSCC cell lines cultured in 3D: (**A**) FaDu, (**B**) SCC-152, (**C**) Detroit-562. Mean values from three biological experiments, with five technical repeats per experiment, are shown ±SD.

Overall, the tested KDM inhibitors and cisplatin required substantially higher concentrations to effectively reduce spheroid viability compared to 2D cultures (Figure 6). An IC50 value for GSK-J4 could only be determined in FaDu spheroids (IC50 = 32.1 µM), whereas in SCC-152 and Detroit-562 spheroids, the response plateaued before reaching a 50% viability reduction. ML324 showed the greatest potency in FaDu spheroids (IC50 = 12.2 µM), followed by SCC-152 spheroids (IC50 = 24.1 µM). However, in Detroit-562 spheroids, the viability-reducing effect of ML324 reached a plateau at approximately 10 µM (~35–40% viability reduction). Consistent with observations under 2D conditions, JIB-04 proved to be the most effective agent in reducing the viability of FaDu (IC50 = 5.1 µM) and SCC-152 (IC50 = 4.7 µM) spheroids. However, in Detroit-562 spheroids, JIB-04 effects also plateaued near 10 µM (~35% reduction), failing to lower viability below 50%. Notably, cisplatin maintained concentration-dependent viability reduction across all three spheroid lines, yielding IC50 values of 13.8 µM (SCC-152), 15.5 µM (FaDu), and 30.2 µM (Detroit-562).

### 3.6. Pre-Treatment of HNSCC Spheroids with Inhibitors of KDM4–6 Improves Cisplatin-Mediated Cell Viability Reduction

**Figure 7:**
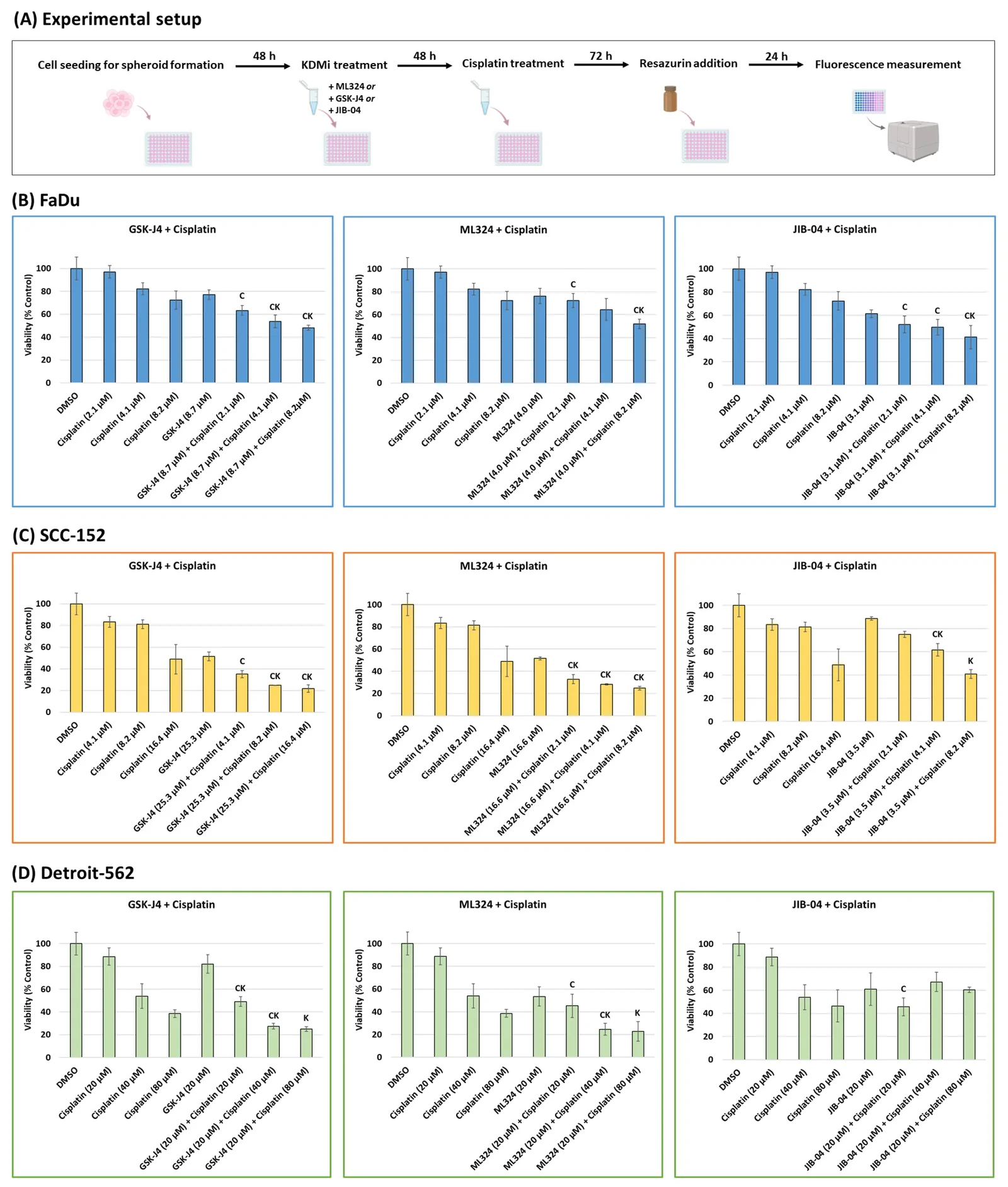
The effect of KDM inhibitors (ML324, GSK-J4, JIB-04) pre-treatment, followed by treatment with cisplatin, on cell viability in HNSCC cell lines cultured in 3D. (**A**) The schematic diagram of the experimental setup, (**B**) results in FaDu spheroids, (**C**) SCC-152 spheroids, (**D**) Detroit-562 spheroids. Mean values from three biological experiments, with six technical repeats per experiment, are shown ±SD. The letter “C” indicates a statistically significant difference between the indicated sequential treatment and cisplatin alone, whereas “K” indicates a statistically significant difference between the sequential treatment and the corresponding KDM inhibitor alone, *p* < 0.05.

Based on the pre-treatment model of KDMi implementation tested in monolayer cell culture (Figure 5), we conducted a similar treatment in spheroid cell cultures (Figure 7). The primary aim was to identify the combinations of KDMi and cisplatin that would enhance the reduction in cell viability compared to cisplatin alone.

In all cell lines, we observed an increase in cisplatin activity following KDMi pre-treatment (marked as C in Figure 7). For GSK-J4, this effect was found independent of cisplatin concentration, except at the highest concentration tested in Detroit-562 spheroids. Furthermore, ML324 showed a similar efficacy profile to GSK-J4, with two exceptions: at the intermediate cisplatin concentration in FaDu spheroids and at the highest concentration in Detroit-562 spheroids. In turn, significant results for JIB-04 were limited to FaDu spheroids and selected cisplatin concentrations in the other cell lines (the intermediate concentration in SCC-152 spheroids and the lowest concentration in Detroit-562 spheroids).

The enhancement in the activity of the combinations of KDMi and cisplatin in comparison to either of the individually applied chemicals (indicated by the “CK” symbol in Figure 7) was another beneficial effect observed. Among all KDMi and cell lines, only in JIB-04 in Detroit-562 spheroids was this result not detected at any cisplatin concentration.

### 3.7. Co-Treatment of HNSCC Spheroids with Inhibitors of KDM4–6 and Cisplatin Is Less Effective Compared to Pre-Treatment

**Figure 8:**
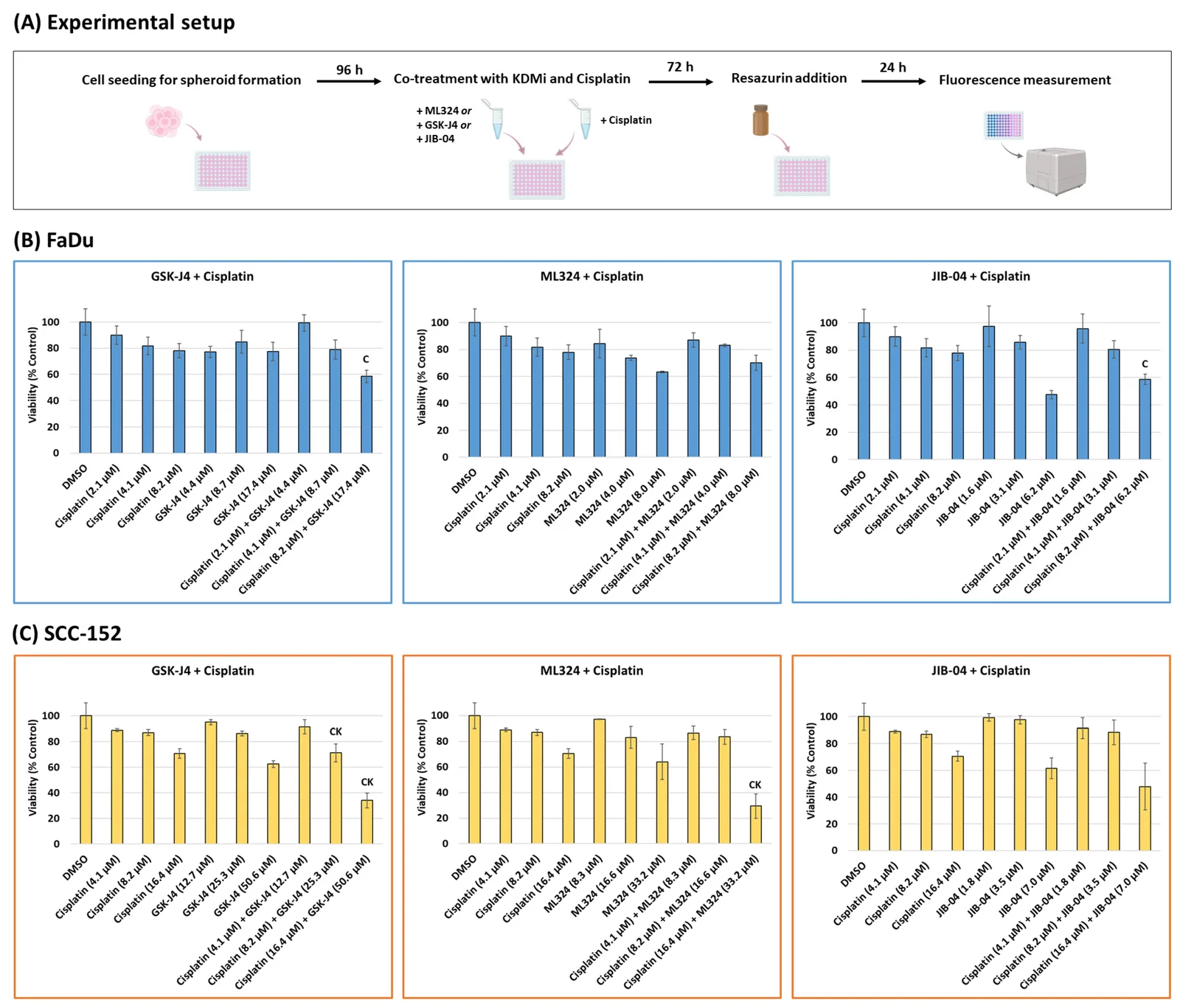
The effect of KDM inhibitors (ML324, GSK-J4, JIB-04) co-treatment with cisplatin, on cell viability in HNSCC cell lines cultured in 3D: (**B**) FaDu, (**C**) SCC-152,. The experimental setup is presented in panel (**A**). Mean values from three biological experiments, with six technical repeats per experiment, are shown ±SD. The letter “C” indicates a statistically significant difference between the indicated sequential treatment and cisplatin alone, whereas “K” indicates a statistically significant difference between the sequential treatment and the corresponding KDM inhibitor alone, *p* < 0.05.

In our previous study, we showed that co-treatment of cells under 2D conditions with cisplatin and KDM inhibitors did not result in enhanced cytotoxic effects in FaDu and SCC-040 cell lines [21]. In the current study, we sought to validate this finding using a more physiologically relevant 3D spheroid model (Figure 8). Indeed, we observed that the concurrent treatment of FaDu spheroids with cisplatin and KDMi did not lead to significantly greater viability reduction compared with treatment with the respective KDMi alone (Figure 8B). However, in SCC-152 spheroids, combined treatment with GSK-J4 and cisplatin at moderate and highest tested concentrations resulted in significantly greater viability reduction than treatment with either compound alone. Similarly, concurrent treatment of SCC-152 spheroids with ML324 and cisplatin at the highest tested concentrations significantly enhanced the reduction in viability compared with the respective single-agent treatments. The combination of JIB-04 with cisplatin did not yield additional beneficial effects at the tested concentrations (Figure 8C).

### 3.8. HNSCC Cells Subjected to Prolonged Cisplatin Pre-Treatment Display Altered Sensitivity to KDM Inhibitors

**Figure 9:**
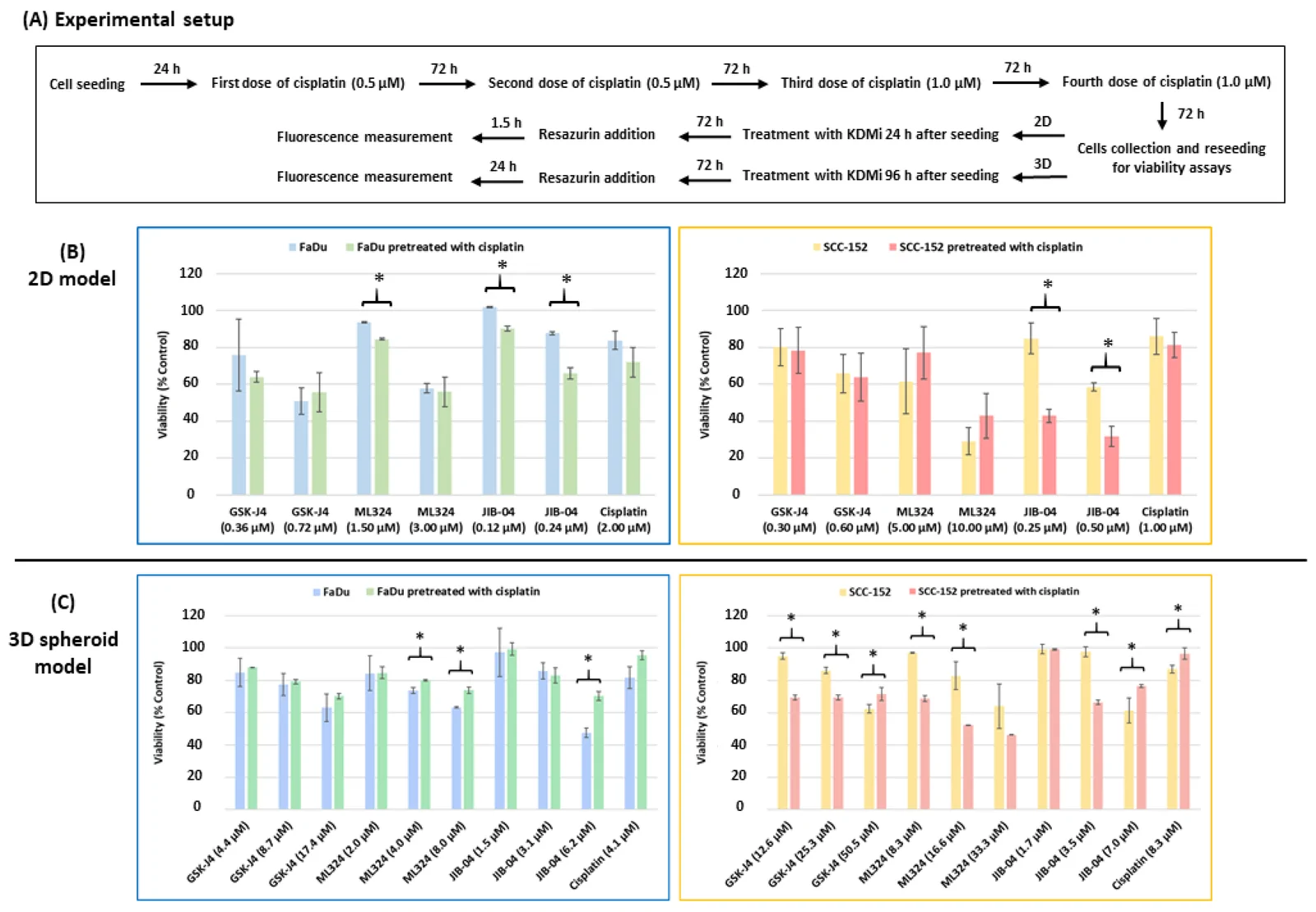
The effect of KDM inhibitors (ML324, GSK-J4, JIB-04), and cisplatin on viability of FaDu and SCC-152 cells subjected to prolonged pretreatment with cisplatin in 2D (**B**) and 3D spheroid model (**C**). The experimental setup is presented in panel (**A**). Mean values from at least two biological experiments, with at least three technical repeats per experiment, are shown ±SD. An asterisk (*) above the bar indicates a statistically significant difference compared with the DMSO control (viability = 100%), * *p* < 0.05.

Persister cells remaining after chemotherapy pose a threat to long-term positive outcomes because of their potential for leading to recurrence [34]. Indeed, recurrence is one of the factors responsible for treatment failure in advanced HNSCC patients [7]. Thus, the eradication of persister cells is a strategy which aims at decreasing the risk of recurrence. Therefore, we also evaluated whether KDMi treatment could also be effective against cells exposed to prolonged pre-treatment with cisplatin. To investigate this, we employed an experimental setup in which FaDu and SCC-152 cells received a total of four doses of cisplatin and were subsequently collected and reseeded for viability assays under both 2D and 3D conditions, followed by treatment with KDMi (Figure 9A). Although this experimental setup did not markedly alter the cells’ sensitivity to cisplatin, it affected their response to KDMi treatment. Under 2D conditions, both FaDu and SCC-152 cells exhibited increased sensitivity to JIB-04 treatment (Figure 9B). However, this effect was not observed in FaDu cells grown as spheroids (Figure 9C). On the other hand, SCC-152 spheroids were more sensitive to KDMi treatment, although only at the lower concentration tested. These results imply that KDMi could affect the survival of cisplatin-persister cells.

## 4. Discussion

Besides genetic mutations, epigenetic alterations are significantly implicated in driving neoplastic transformation [35]. Apart from changes in the profile of DNA methylation, changes in chromatin structure and function can be mediated by altering the pattern of histone modifications. Blocking the deregulation of the histone methylation profile showed the capacity of preventing bladder or prostate tumor formation in experimental animals [36,37]. Moreover, the formation of oral tumors induced by exposure to carcinogenic 4-nitroquinoline 1-oxide could be diminished by the genetic deletion or pharmacological inhibition of LSD1 histone demethylase in mice [38]. Evidence shows that epigenetic writers and erasers which are responsible for histone methylation/demethylation offer a promising anti-cancer therapeutic strategy [39], also in HNSCC [13,40].

### 4.1. KDMs Are Overexpressed in HPV-Positive and -Negative HNSCC Cases

Histone lysine demethylases from the Jumonji C family (KDM2–8) require Fe^2+^, O_2_, and 2-ketoglutarate to remove methyl groups from histone lysine residues. KDMs are residue-specific, and KDM4, KDM5, and KDM6 lead to the demethylation of H3K9me2/3, H3K4me2/3, and H3K27me2/3, respectively [12,41]. Acting within the network of many epigenetic proteins, they are implicated in the regulation of transcriptional programs and other cellular functions, e.g., DNA repair or stemness [14,39,40]. Importantly, we presented evidence that the majority of these KDMs were upregulated in HNSCC patients from the TCGA cohort (although the difference was not statistically significant in the case of *KDM4C* and *KDM6A*) with the single exception of *KDM5D*. Interestingly, *KDM5D* was strongly associated with longer overall survival of HNSCC patients, irrespective of HPV status. This suggests that KDM5D may stand out regarding its role in HNSCC carcinogenesis, but this should be tested further to allow more evidence-supported conclusions to be drawn. So far, *KDM5D* has been suggested to act as a tumor suppressor in colorectal and lung cancers, at least in part via histone-independent mechanisms [42,43]. *KDM5D* is located on the Y chromosome, and a recent study showed that chemically induced oral carcinogenesis was associated with decreased expression of Y chromosome genes. It was shown that Y-linked loss of *KDM5D* could contribute to carcinogenesis by upregulated interleukin-17 signaling [44].

Etiologically, HNSCC can be stratified into HPV-independent and HPV-dependent tumors, which is associated with important clinical consequences related to the choice of treatment and clinical prognosis [3,8]. While the exact epigenetic landscape may differ between HPV-negative and HPV-positive HNSCC cases [40], it seems that KDM inhibition may be an eligible therapeutic strategy in both subgroups of patients. We have shown that most KDM4–6 demethylases were upregulated in HNSCC patients from the TCGA cohort irrespective of HPV status. Although we used only one HPV-positive HNSCC cell line (SCC-152) in our study, we observed that these cells showed comparable sensitivity towards the analyzed KDMi to the HPV-negative cell lines. However, broader studies would be required to more comprehensively validate the significance of HPV-stratification for the evaluation of sensitivity to KDM inhibition.

### 4.2. KDM4–6 Inhibitors Reduce Viability in 2D- and 3D-Cultured HNSCC Cells

In this study, we compared the activity of selective inhibitors of KDM4 and KDM6 (ML324 and GSK-J4, respectively), and a non-selective inhibitor of KDM4–6 demethylases (JIB-04). We have shown that all three inhibitors significantly reduced cell viability in all the studied cell lines (SCC-152, Detroit-562, UM-SCC-1). Interestingly, JIB-04 showed potent activity at the lowest concentrations. In our previous studies, we have shown that the analyzed KDMi effectively reduced cell viability also in other HNSCC cell lines cultured in 2D (FaDu, CAL 27, SCC-040) [21,30,45]. Moreover, we have shown that ML324, GSK-J4, and JIB-04 could mildly promote apoptosis in FaDu, CAL 27, SCC-040, and SCC-152 cells [21,30,45]. In this study, we extended our analyses and performed viability assessment in cells grown in 3D as spheroids. We observed that higher concentrations of all three KDMi were required to acquire equipotent reduction in cell viability. This is, however, not surprising [46]. Importantly, the compounds were active against spheroids of FaDu and SCC-152 cell lines, but Detroit-562 spheroids were more resistant to KDMi action. Again, of all three analyzed KDMi, in general JIB-04 showed the highest potency at the lowest concentrations. Although these chemicals have been rarely studied in HNSCC cells so far, our results are in concert with other scientific findings. KDM4A promoted oral cancer cell proliferation and avoidance of apoptosis, and its knockdown reduced xenograft tumor growth [47]. Moreover, KDM4C silencing also reduced HNSCC xenograft tumor growth in experimental mice [48]. Furthermore, KDM5B promoted cell proliferation, and its knockdown reduced HNSCC growth in vitro and in vivo [49]. With respect to other cancer types, GSK-J4 was shown to reduce cell viability, proliferation, and xenograft tumor growth in prostate and cervical cancers, or osteosarcoma and retinoblastoma [50,51,52,53,54]. On the other hand, JIB-04 reduced the viability, migration, and invasion of breast and hepatocellular cancer cells, and diminished breast cancer xenograft tumor growth [55,56].

### 4.3. KDM4–6 Inhibitors Affect the Expression of Pluripotency Genes in HNSCC Cells

The mechanisms of the anti-cancer activity of KDM inhibitors are diverse, and they are associated with the modulation of cell proliferation, DNA repair, apoptosis, cell motility, cellular cross-talk, or immune modulation [14]. Importantly, the involvement in the regulation of stemness phenotype may significantly contribute to their action. Indeed, KDM4C inhibition using luteolin significantly reduced the protein levels of SOX2, OCT4, and NANOG, and ALDH1 activity in ovarian cancer cells [57]. Moreover, *KDM4C* knockdown reduced the percentage of ALDH-positive esophageal cancer cells [58]. KDM5B was associated with increased expression of stemness markers (OCT4, NANOG, ALDH1A) in HNSCC cells [59,60], and a KDM5 inhibitor—CPI-455—reduced the expression of stemness markers in oral cancer cell lines [61]. In addition, KDM6B was shown to transcriptionally regulate *SOX2* expression [62]. Furthermore, the KDM4–6 inhibitor, JIB-04, was shown to reduce the expression of several stemness markers (*CD133/PROM1*, *CD44*, *LGR5*, *EpCAM*) in hepatocellular cancer cells [56]. We have previously shown that ML324, GSK-J4 and JIB-04 reduced the expression of *ALDH1A1*, *ABCG2*, *POU5F1*, and *SOX2* in FaDu, and, to a lesser extent, SCC-152 cells [30]. In this study, we provided further evidence for the ability of ML324 and JIB-04 to reduce the level of expression of *ALDH1A1* and *SOX2*, and to reduce SOX2 protein level, in metastatic pharyngeal carcinoma Detroit-562 cells. Interestingly, the inhibitors induced the expression of *ABCG2* and *NANOG* in this cell line, which points to the complexity of the regulation of transcriptional programs related to stemness. Nevertheless, these data corroborate that the regulation of stemness potential is an important factor in KDM inhibition. This is also supported by the correlation between the expression of KDMs and stemness markers in HNSCC patients, which we have shown in this paper. Indeed, KDM5A, KDM6A, and KDM6B were recently shown to be engaged in the transcriptional regulation of the expression of stemness markers—*POU5F1*, *SOX2*, and *NANOG* [63,64]. In line with this view, we have previously shown that ML324, GSK-J4, and JIB-04 potently reduced the clonogenic potential of FaDu and SCC-152 cells [30].

### 4.4. Enhancement of Cisplatin-Mediated Effects After Epigenetic Priming with KDM Inhibitors

One of the main aims of this study was the validation of the hypothesis that epigenetic priming with KDM4–6 inhibitors could sensitize HNSCC cells to cisplatin. The inclusion of chemotherapy is indicated in the treatment of locoregionally advanced, recurrent, metastatic, or unresectable head and neck tumors. Advanced HNSCC is associated with a high risk of relapse, which significantly reduces prognosis and patient survival [65]. Thus, novel treatments, including novel targeted drugs or novel drug combinations that could exert enhanced potency, are necessary to improve outcomes in this group of HNSCC patients. We recently analyzed whether concurrently applied KDM4–6 inhibitors could increase the effects of cisplatin in HNSCC cells, and found that the studied KDMi did not synergize with cisplatin unless a triple combination with olaparib, a PARP1 inhibitor, was used. None of the inhibitors enhanced cisplatin-mediated apoptosis induction following a concurrent incubation for 48 h [21]. In this study, we have shown that the co-treatment of FaDu cell spheroids with cisplatin and the studied KDMi did not offer an advantage over individual treatments, which is similar to our previous findings from 2D culture [21]. However, some enhanced response upon co-treatment with cisplatin and GSK-J4 or ML324 was observed in SCC-152 cell spheroids. On the other hand, several previous reports pointed to the possibility of so-called “epigenetic priming”, where the initial exposure to epigenetic modulators could promote the effects of subsequently applied drugs [22,23]. We have shown that all three studied KDMi exhibited at least some potential for cisplatin sensitization, which was cell line- and concentration-dependent. These observations are in line with findings of other authors. The silencing of KDM4A or KDM4C led to the sensitization of ovarian or hepatoma cancer cells to cisplatin [66,67]. The overexpression of KDM5B resulted in the development of cisplatin resistance in nasopharyngeal cancer cells [68]. Moreover, GSK-J4 was shown to enhance the cytotoxic potential of cisplatin in osteosarcoma and testicular cancer cells [19,69]. In addition, ML324 and JIB-04 sensitized lung cancer cells to cisplatin, and it was shown that JIB-04 mediated these effects by modulating DNA repair via homologous recombination [18].

### 4.5. Sensitivity of Cisplatin-Persister HNSCC Cells to KDM4–6 Inhibitors

In our study, we also evaluated a reversed sequential treatment, in which cells were exposed to prolonged cisplatin treatment first. The protocol of the extended exposure of cells to cisplatin over the period of two weeks, with the application of four doses of the drug, did not lead to the development of cisplatin tolerance in FaDu or SCC-152 cells, as evidenced by cell viability data. FaDu cells exhibited the same response to GSK-J4, irrespective of prior treatment with cisplatin. On the other hand, JIB-04, and to a lesser extent ML324, were slightly more potent towards cisplatin-treated cells, but only in 2D cell culture. More pronounced effects were observed in SCC-152 cells exposed to the extended treatment with cisplatin. JIB-04 led to significant enhancement of viability reduction in the 2D model, but the effects were weaker in 3D-grown spheroids. On the other hand, ML324 and GSK-J4 more potently reduced the viability of cisplatin pre-treated Detroit-562 cells only in the 3D model. These results suggest that the inhibition of KDM4-6 could be a viable anti-cancer strategy in patients who were previously treated with cisplatin,. However, it may not be universally active, and further research should focus on the establishment of predictors of such effects.

Our reported findings are based only on the results of cell viability assessment, which is a limitation of this study. Further comprehensive analyses should be performed to extensively validate the epigenetic priming hypothesis.

## 5. Conclusions

We have shown that KDM4–6 demethylases, with the exception of KDM5D, are upregulated in HNSCC, and they constitute promising therapeutic targets. Individually applied inhibitors of KDM4 (ML324), KDM6 (GSK-J4), and KDM4–6 (JIB-04) potently reduced viability in 2D and 3D cultures of HNSCC cells. Moreover, the pre-treatment of cells with the studied KDMi to some extent sensitized HNSCC cells to cisplatin. These data provide some evidence that KDM4–6 could be applied in HNSCC treatment both individually, and in combination with cisplatin. Further comprehensive studies should focus on the detailed explanation of the mechanism of KDMi action in HNSCC cells, and the validation of exact indications for their use.

## Figures and Tables

**Table 1:** The results of univariate analysis of the association between low level (*n* = 258) versus high level (*n* = 258) KDM expression and overall survival (OS) in HNSCC patients using the GEPIA3 tool. Statistically significant (*p* < 0.05) difference in hazard ratio (HR) is marked in bold.

Gene	Median OSLow Expression	Median OS High Expression	χ^2^	*p*-Value	HR (95% CI)	*p* (HR)
*KDM4A*	49.41	57.43	5.14	**0.0233**	0.74 (0.56~0.96)	**0.0238**
*KDM4B*	46.98	57.89	4.32	**0.0377**	0.76 (0.58~0.99)	**0.0384**
*KDM4C*	60.39	54.90	0.18	0.67	1.06 (0.81~1.38)	0.67
*KDM4D*	54.90	57.89	0.22	0.641	0.94 (0.72~1.23)	0.64
*KDM5A*	54.90	56.44	0.02	0.886	1.02 (0.78~1.33)	0.887
*KDM5B*	56.90	53.91	0.14	0.712	0.95 (0.73~1.24)	0.712
*KDM5C*	56.90	56.44	0.05	0.816	1.03 (0.79~1.35)	0.816
*KDM5D*	35.45	67.81	12.23	**0.00047**	0.62 (0.47~0.81)	**0.00053**
*KDM6A*	49.41	57.89	1.96	0.161	0.83 (0.63~1.08)	0.162
*KDM6B*	60.39	49.41	0.35	0.552	1.08 (0.83~1.42)	0.552

**Table 2:** The results of the analysis of the correlation between KDM4-6 expression and stemness markers (*ALDH1A1*, *ABCG2*, *SOX2*, *POU5F1*, *NANOG*) in (**A**) HNSCC patients (*n* = 520) and (**B**) HPV-negative (*n* = 421) or (**C**) HPV-positive (*n* = 97) HNSCC cases performed using TIMER3. Statistically significant (*p* < 0.05) positive correlation (Spearman’s rho > 0) is marked with green bold, while statistically significant (*p* < 0.05) negative correlation (Spearman’s rho < 0) is marked in red bold.

**(A) HNSCC**	***KDM4A***	***KDM4B***	***KDM4C***	***KDM4D***	***KDM5A***	***KDM5B***	***KDM5C***	***KDM5D***	***KDM6A***	***KDM6B***
*ALDH1A1*	**0.287**	**0.364**	**0.186**	**0.17**	**0.291**	0.053	**0.157**	**0.16**	**0.231**	**0.208**
*ABCG2*	**0.21**	**0.126**	**0.196**	**0.242**	**0.328**	**0.116**	**0.248**	0.034	**0.245**	**0.196**
*SOX2*	**0.369**	**0.443**	**0.241**	**0.222**	**0.307**	0.058	**0.203**	**0.136**	**0.297**	**0.202**
*POU5F1*	**0.296**	**0.231**	**0.231**	**0.285**	**0.135**	−0.054	**0.102**	**0.212**	**0.24**	0.042
*NANOG*	**0.138**	**0.178**	**0.114**	0.071	**0.167**	0.009	**0.092**	**0.128**	**0.129**	**0.13**
**(B) HNSCC** **HPV-negative**	***KDM4A***	***KDM4B***	***KDM4C***	***KDM4D***	***KDM5A***	***KDM5B***	***KDM5C***	***KDM5D***	***KDM6A***	***KDM6B***
*ALDH1A1*	**0.271**	**0.361**	**0.149**	**0.173**	**0.3**	**0.121**	**0.18**	**0.111**	**0.227**	**0.242**
*ABCG2*	**0.22**	**0.139**	**0.204**	**0.282**	**0.286**	0.08	**0.219**	0.035	**0.241**	**0.175**
*SOX2*	**0.32**	**0.399**	**0.173**	**0.183**	**0.321**	**0.189**	**0.209**	0.04	**0.238**	**0.267**
*POU5F1*	**0.291**	**0.2**	**0.14**	**0.259**	**0.167**	0.051	**0.125**	**0.121**	**0.182**	**0.142**
*NANOG*	**0.139**	**0.182**	**0.096**	0.051	**0.156**	0.061	**0.1**	**0.106**	**0.1**	**0.165**
**(C) HNSCC** **HPV-positive**	***KDM4A***	***KDM4B***	***KDM4C***	***KDM4D***	***KDM5A***	***KDM5B***	***KDM5C***	***KDM5D***	***KDM6A***	***KDM6B***
*ALDH1A1*	**0.28**	**0.307**	**0.211**	0.065	**0.231**	−0.077	0.007	**0.273**	0.119	0.135
*ABCG2*	**0.235**	0.144	**0.215**	0.158	**0.51**	**0.221**	**0.409**	0.096	**0.335**	**0.291**
*SOX2*	**0.475**	**0.465**	**0.33**	0.175	**0.318**	−0.136	0.166	**0.276**	**0.347**	0.143
*POU5F1*	0.115	0.034	**0.351**	0.163	−0.034	**−0.227**	−0.017	**0.246**	**0.239**	−0.177
*NANOG*	0.082	0.122	0.143	0.058	0.194	−0.139	0.058	0.177	0.18	0.022

## Data Availability

The datasets generated and analyzed during the current study are available from the corresponding author on reasonable request.

## Supplementary Materials

The following supporting information can be downloaded at: https://www.mdpi.com/article/10.3390/genes17091047/s1, File S1: KDM4–6 expression in HNSCC patients; File S2: The association between KDM4–6 expression and overall survival in HNSCC patients; Figure S1: STRING analysis of the protein network of KDM4-6 proteins and pluripotency factors; Figure S2: Optimization of spheroid growth conditions.

## Abbreviations

The following abbreviations are used in this manuscript:

HNSCC	Head and neck squamous cell carcinoma
KDM	Histone lysine demethylase
KDMi	Histone lysine demethylase inhibitor
TCGA	The Cancer Genome Atlas
OS	Overall survival
HPV	Human papilloma virus
