# Supplementary material for: The Inhibition of Histone Lysine Demethylases Affects Head and Neck Cancer Cell Growth and Response to Cisplatin
Source: Genes (Basel). 2026 Aug 30;17(9):1047. doi: 10.3390/genes17091047 (PMC13606303; doi:10.3390/genes17091047)
Supplement: Supplementary file 1 [file genes-17-01047-s001.zip › Supplementary Figure S1.pdf]

Dorna et al. The Inhibition of Histone Lysine Demethylases Affects Head and Neck Cancer Cell Growth and Response to Cisplatin.

Supplementary Figure S1.

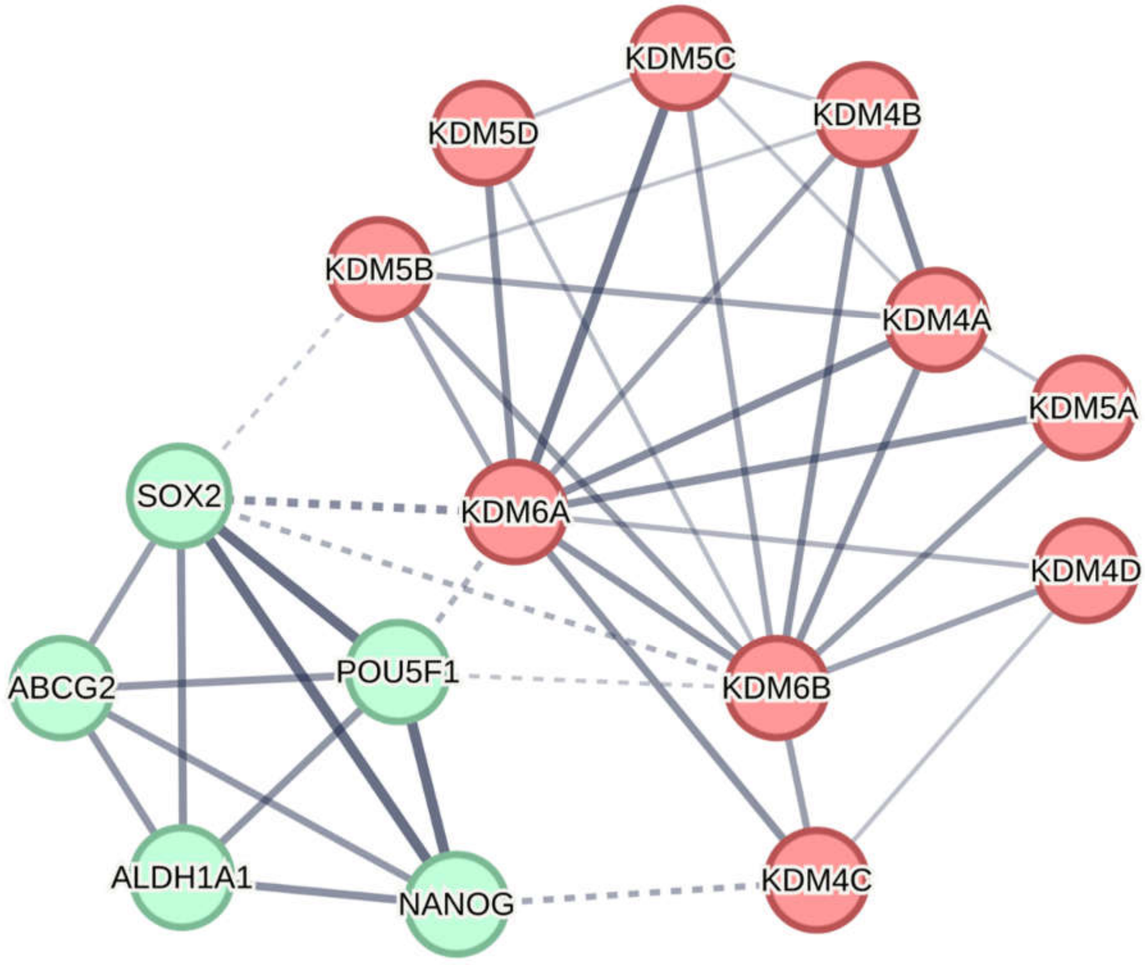

Figure S1. STRING analysis of the protein network of KDM4-6 proteins (red), and pluripotency factors (green: ABCG2, ALDH1A1, SOX2, POU5F1, NANOG), in human cells. Clusters (red and green) were identified with k-means legacy algorithm. Interactions between proteins from separate clusters are shown in dotted lines, and interactions between proteins within the same cluster are shown with solid lines. Lowest thickness of line edges notifies medium confidence of evidence for protein association (0.4), while intermediate and highest thickness of lines notify high (0.7), and highest (0.9) strength of evidence for the association between proteins, respectively.
