# Supplementary material for: The Inhibition of Histone Lysine Demethylases Affects Head and Neck Cancer Cell Growth and Response to Cisplatin
Source: Genes (Basel). 2026 Aug 30;17(9):1047. doi: 10.3390/genes17091047 (PMC13606303; doi:10.3390/genes17091047)
Supplement: Supplementary file 1 [file genes-17-01047-s001.zip › Supplementary Figure S2.pdf]

Dorna et al. The Inhibition of Histone Lysine Demethylases Affects Head and Neck Cancer Cell Growth and Response to Cisplatin.

Supplementary Figure S2 – Optimization of spheroid growth conditions.

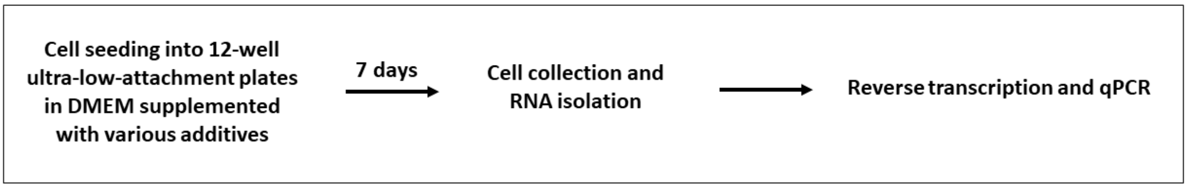

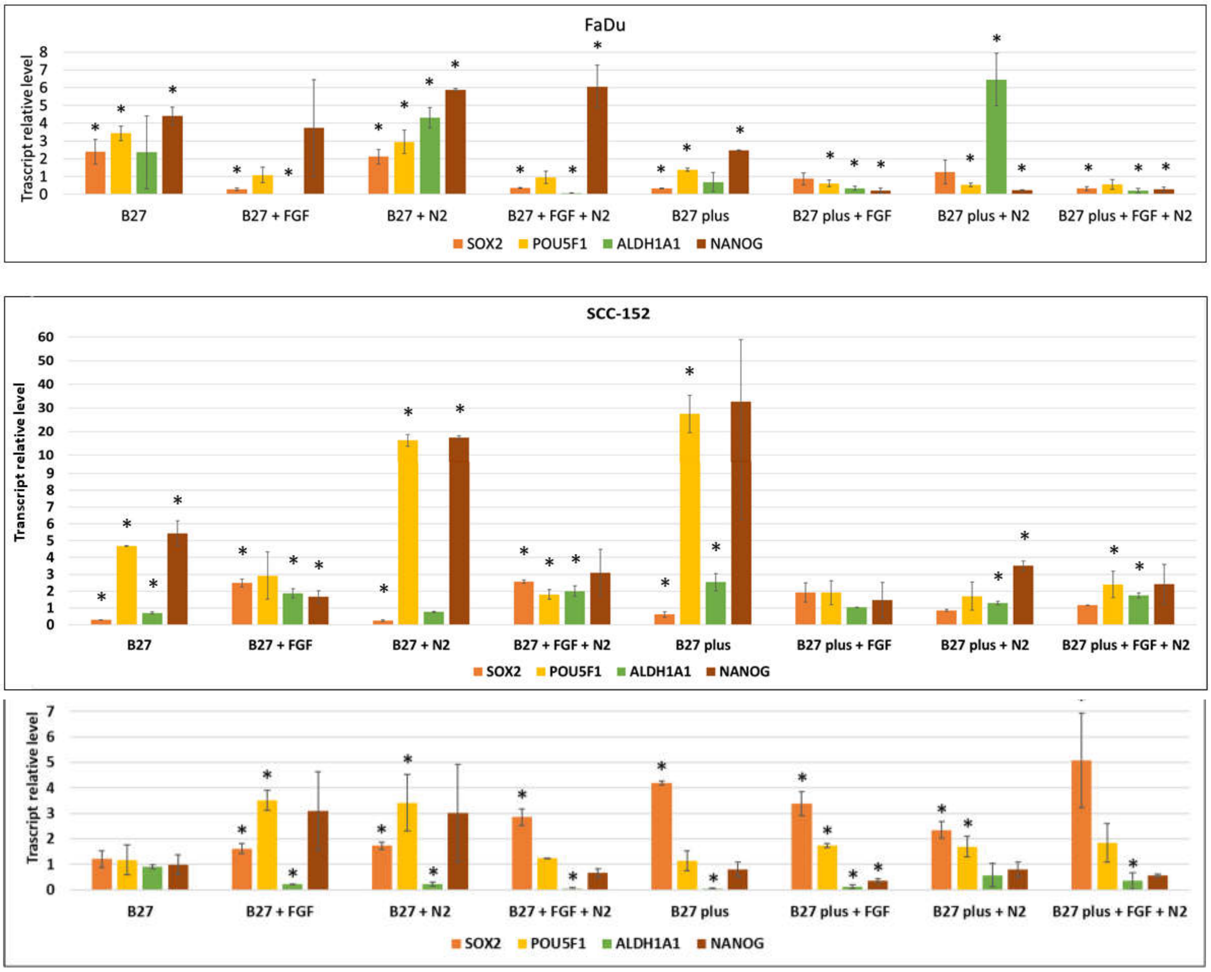

Figure S2. Effect of culture medium supplements on stemness-related gene expression. Relative mRNA expression levels of stemness-related genes in FaDu, SCC-152, and SCC-040 cell lines cultured with various media supplements as indicated on the x-axis. All experimental media were supplemented with 0.5% antibiotics. Data represent mean values from three independent biological experiments ±SD. Asterisks (*) denote statistically significant differences compared to the control group - cells cultured in DMEM supplemented with 5% FBS and 1% antibiotics (transcript level = 1), *$p < 0.05$
